# The role of emergency medical service providers in the decision-making process of prehospital trauma triage

**DOI:** 10.1007/s00068-018-1006-8

**Published:** 2018-09-20

**Authors:** Eveline A. J. van Rein, Said Sadiqi, Koen W. W. Lansink, Rob A. Lichtveld, Risco van Vliet, F. Cumhur Oner, Luke P. H. Leenen, Mark van Heijl

**Affiliations:** 1 Utrecht Trauma Centre, University Medical Centre Utrecht, Suite: G04.228, Heidelberglaan 100, 3584 CX Utrecht, The Netherlands; 2 Department of Orthopaedic Surgery, University Medical Centre Utrecht, Utrecht, The Netherlands; 3 Department of Surgery, Elisabeth-TweeSteden Hospital, Tilburg, The Netherlands; 4 Regional Ambulance Facilities Utrecht, RAVU, Bilthoven, The Netherlands; 5 Regional Ambulance Facilities Brabant Central-West-North, ’s Hertogenbosch, The Netherlands; 6 Department of Traumatology, University Medical Centre Utrecht, Utrecht, The Netherlands; 7 Department of Surgery, Diakonessenhuis Utrecht/Zeist/Doorn, Utrecht, The Netherlands

**Keywords:** Trauma, Triage, Compliance, Adherence, Prehospital

## Abstract

**Purpose:**

Severely injured patients should be treated at higher-level trauma centres, to improve chances of survival and avert life-long disabilities. Emergency medical service (EMS) providers must try to determine injury severity on-scene, using a prehospital trauma triage protocol, and decide the most appropriate type of trauma centre. The objective of this study is to investigate the role of EMS provider judgment in the prehospital triage process of trauma patients, by analysing the compliance rate to the protocol and administering a questionnaire among EMS providers.

**Methods:**

All trauma patients transported to a trauma centre in two different regions of the Netherlands were analysed. Compliance rate was based on the number of patients meeting the triage criteria and transported to the corresponding level trauma centre. The questionnaire was administered among EMS providers. Descriptive statistics were used to analyse the data.

**Results:**

For adult patients, the compliance rate to the level I criteria of the triage protocol was 72% in Central Netherlands and 42% in Brabant. For paediatric patients, this was 63% and 38% in Central Netherlands and Brabant, respectively. The judgment on injury severity was mostly based on the injury-type criteria. Additionally, the distance to a level I trauma centre influenced the decision for destination facility in the Brabant region.

**Conclusion:**

The compliance rate varied between regions. Improvement of prehospital trauma triage depends on the accuracy of the protocol and compliance rate. A new protocol, including EMS provider judgment, might be the key to improvement in the prehospital trauma triage quality.

## Introduction

Prehospital trauma triage is of vital importance to ensure transport to a trauma centre with the appropriate level of care for trauma patients. Patients with severe injuries should be treated at higher level trauma centres, to reduce mortality and morbidity. Patients without severe injuries should be transported to a lower level facility, to reduce burden on the higher level trauma centres’ unnecessary costs [1–3].

A prehospital trauma triage protocol is in place to help emergency medical service (EMS) providers discriminate between patients with and without severe injuries, and decide the most appropriate type of trauma centre [4, 5]. The accuracy of a triage protocol is essential, but ultimately it is the EMS provider who determines the destination of the patient. The literature is undecided on the additional value of EMS provider judgment. Previous reports have shown that cognitive reasoning processes contribute to the identification of severely injured patients, potentially missed by triage criteria [6–9]. Others found the judgment of EMS providers to be less accurate [5, 10].

Prehospital trauma triage protocols have been studied extensively over the past decades [11–14]. However, it is currently unknown what factors are associated with EMS provider judgment and to what extent compliance to the triage protocol influences quality of prehospital trauma triage. The objective of this study is to gain insight in the role of EMS providers, in terms of their judgment as well as their reasoning in the prehospital triage process of trauma patients through (1) an analysis of the compliance rate to the triage protocol in a prospectively collected dataset and (2) a survey among EMS providers in two regions of the Netherlands.

## Methods

### Study design

This study consists of two parts: (1) an evaluation of compliance to the prehospital trauma triage protocol in a prospective cohort, and (2) a survey, both performed in two regions of the Netherlands: Central Netherlands and Brabant. The survey was web based and conducted among EMS providers to gain insight on their judgment in the prehospital trauma triage process (“Appendix”). These two regions were chosen because both differ in geographical distance to trauma centres, mechanism of injury and prevalence of severe injury [15].

In the Netherlands, level I trauma centres are designated to provide the appropriate level of care for severely injured patients [16]. Central Netherlands has one level I trauma centre (University Medical Centre Utrecht) and seven level II or III trauma centres. The region covers 535 square miles and serves 1.3 million residents. Brabant has 1 level I trauma centre (Elisabeth-TweeSteden Hospital Tilburg) and 11 level II or III trauma centres. This region covers 1343 square miles and has 1.7 million residents.

**Fig. 1:**
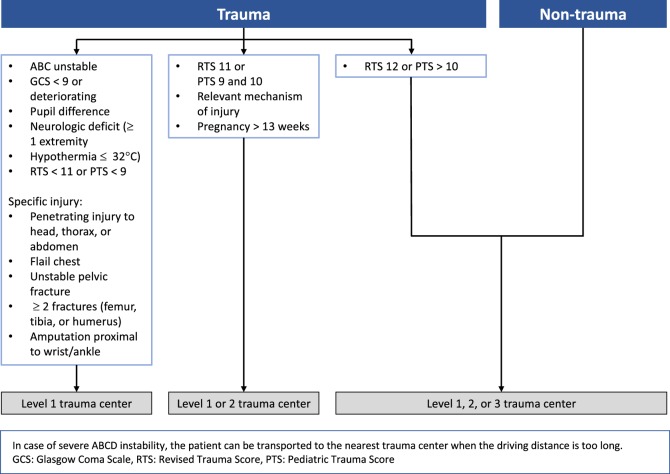
The national field triage protocol of the Netherlands

In the Netherlands, all ambulances are staffed by: an ambulance nurse (in this article referred to as EMS provider), who is skilled and trained in medical knowledge and procedures, and an ambulance driver who is able to assist the EMS provider [17]. The ambulance nurses are registered nurses with additional mandatory 7-month national training in prehospital care, which includes experience in the field and knowledge of the triage protocol. The triage protocol used in the Netherlands, the National Protocol for Ambulance Services (Fig. 1), is based on the Field Triage Decision Scheme established by the American College of Surgeons Committee on Trauma [4, 18].

This study was judged by the Medical Ethical Committee of University Medical Centre Utrecht as not subject to the Medical Research Involving Human Subjects Act.

### Participating trauma patients

All trauma patients transported with highest priority (siren and lights) to trauma centres in one of the two regions were analysed to determine the compliance rate. Patients were included between January 2012 and June 2014 in the Central Netherlands region and between January 2015 and December 2015 in the Brabant region. Patients transported outside of the studied regions were excluded.

### Data collection of trauma patients

For each patient, the EMS providers record all prehospital information in an electronic prehospital report. These reports were prospectively collected and included: patient demographics, vital signs criteria, injury type criteria, mechanism of injury criteria and initial receiving hospital.

Hospital data were collected from the institutional trauma registry and electronic medical records. The Dutch National Trauma Database registered receiving hospital, Abbreviated Injury Scale (AIS) and mortality for all patients admitted to a hospital. For Central Netherlands, data were also extracted from the electronic patient documentation for patients discharged directly from the emergency department (ED). The injuries were recorded using the Abbreviated Injury Scale (AIS) 1990, Update 1998 and coded by trained data managers after discharge or 30 days after admission. In Brabant, the AIS 2005, update 2008, was used. The data managers were blinded for triage criteria positivity. To determine injury severity, the Injury Severity Score (ISS) was calculated based on the AIS scores.

### Questionnaire and recruitment

The questionnaire focussed on: factors influencing the triage decision, timing of destination decision and possible reasons for, and consequences of undertriage and overtriage. The questions were formulated based on previous research and consensus among the authors. To capture the agreement, the questions were based on a 5-point Likert scale ranging from 1 (factor has no influence) to 5 (factor is highly influential). To get a more accurate understanding of the rationale in the destination decision process, a free text section was included in yes/no questions and at the end for any general comments. The data managers of the specific region sent the 150 EMS providers of Central Netherlands and 220 EMS providers of Brabant a weblink to the questionnaire. A reminder was sent after 4 weeks.

### Outcomes and definitions

For both regions, the compliance rates to the whole triage protocol and the level I criteria were determined for paediatric (< 16 years old) and adult (≥ 16 years old) patients separately. The compliance rate was calculated as

$${\text{Whole protocol}}=\frac{{{\text{Patients meeting triage criteria, transported to the corresponding level trauma center}}}}{{{\text{Patient meeting one or more triage criteria}}}},$$

$${\text{Level I criteria}}=\frac{{{\text{Patients meeting level I criteria, transported to a level I trauma center}}}}{{{\text{Patients meeting one or more level I criteria}}}}.$$

A severely injured patient was defined as a patient with an ISS > 15.

### Missing data

Multiple imputation for missing prehospital variables was used for both regions separately, to calculate the compliance rate. Missing values were predicted based on all other predictors, as well as ISS. In the database of Brabant, the paediatric trauma score was missing in the paediatric patients and the ISS was available for admitted patients only. An ISS < 15 was assumed for patients discharged from the ED, as it has previously been shown all discharged patients had an ISS < 15 in Central Netherlands [19]. The Revised Trauma Score [20] was based on the multiply imputed Glasgow Coma Scale, systolic blood pressure and respiratory rate for both regions.

### Statistical analysis

The data were analysed using descriptive statistics. The response to the questions of the questionnaire was anonymous and the data were managed by data managers. The questions based on the 5-point Likert scale allowed detection of the presence and degree of influence for certain factors on EMS provider judgment in the triage process. Three months after the questionnaire was sent the data of the questionnaires were assessed. All statistical analyses were performed using SPSS v 24.0.

## Results

### Compliance rate

#### Central Netherlands region

In Central Netherlands, 4950 adults and 594 paediatric trauma patients were transported with highest priority to a trauma centre by EMS providers (Table 1). In total, 435 (8.8%) of the adult patients and 26 (4.4%) of the paediatric patients were severely injured (ISS > 15).

**Table 1:** Baseline patient characteristics in Central Netherlands and Brabant regions

Variables	Central Netherlands ≥ 16 years old (*n* = 4950)	Central Netherlands < 16 years old (*n* = 594)	Brabant ≥ 16 years old (*n* = 6859)	Brabant < 16 years old (*n* = 976)
	Mean (SD)	Mean (SD)	Mean (SD)	Mean (SD)
Age (years)	47 (21.3)	9 (4.7)	51 (22.1)	8 (5.0)
ISS	5 (7.1)	4 (5.1)	–	–

Variables	Central Netherlands ≥ 16 years old (*n* = 4950)	Central Netherlands < 16 years old (*n* = 594)	Brabant ≥ 16 years old (*n* = 6859)	Brabant < 16 years old (*n* = 976)
	Number (%)	Number (%)	Number (%)	Number (%)
Male gender	2887 (58.3)	331 (55.7)	3583 (52.2)	223 (61.4)
ISS > 15	435 (8.8)	26 (4.4)	165 (2.4)	2 (0.2)
Destination				
Level I trauma centre	1724 (34.8)	287 (48.3)	1882 (27.4)	300 (30.7)
Level II trauma centre	1326 (26.8)	163 (27.4)	4208 (61.4)	563 (57.7)
Level III trauma centre	1900 (41.2)	144 (24.2)	769 (26.9)	113 (11.6)
Admission to hospital	2039 (41.2)	68 (11.4)	1842 (26.9)	363 (37.2)
In-hospital death	63 (1.3)	1 (0.2)	57 (0.8)	0 (0)

Brabant region: ISS was only available for patients who were admitted or died before admission. Gender missed in 858 (12.5%) adult patients and in 613 (62.8%) paediatric patients

*SD* standard deviation, *ISS* Injury Severity Score

The compliance rate to the whole triage protocol was 72.6% for adult trauma patients (Table 2). The compliance rate to the level I triage criteria for the adult trauma patients was 72.4%. Only 36.3% of the severely injured adult patients met one or more level I triage criteria. Still, 78.4% of the severely injured adult patients were transported to a level I trauma centre. Among the severely injured patients not meeting any of the level I criteria, 67.5% were transported to a level I trauma centre. The compliance rate was lower for elderly patients (> 75 years old): 61.6%, compared to 73.5% for young adults (16–75 years old).

**Table 2:** Distribution of adult trauma patients

Region	Criteria	Level I	Level II	Level III
Central-Netherlands (*n* = 4950)	LPA level I criteria	357 (72.4)	54 (11.0)	82 (16.7)
	ISS > 15	155 (98.1)	1 (0.6)	2 (1.3)
	LPA level I or II criteria	503 (53.6)	179 (19.1)	257 (27.4)
	ISS > 15	126 (91.3)	6 (4.3)	6 (4.3)
	Vital sign level I criteria	207 (72.6)	31 (10.9)	48 (16.8)
	ISS > 15	113 (97.4)	1 (0.9)	2 (1.7)
	Vital sign level I or II criteria	136 (52.7)	40 (15.5)	82 (31.8)
	ISS > 15	50 (92.6)	2 (3.7)	3 (5.6)
	Injury-type level I criteria	200 (76.9)	25 (9.6)	35 (13.5)
	ISS > 15	81 (100)	0 (0)	0 (0)
	Injury-type level I or II criteria	26 (45.6)	23 (40.4)	8 (21.6)
	ISS > 15	9 (100)	0 (0)	0 (0)
	Mechanism of injury level I or II criteria^a^	369 (54.5)	137 (20.2)	171 (25.3)
	ISS > 15	79 (91.9)	4 (4.7)	3 (3.5)
Brabant (*n* = 6859)	LPA level I criteria	213 (41.8)	249 (48.8)	48 (9.4)
	ISS > 15	53 (89.8)	5 (8.5)	1 (1.7)
	LPA level I or II criteria	174 (29.9)	346 (59.6)	61 (10.5)
	ISS > 15	27 (77.1)	6 (17.1)	3 (8.6)
	Vital sign level I criteria	179 (42.8)	201 (48.1)	39 (9.3)
	ISS > 15	50 (92.6)	3 (5.6)	1 (1.9)
	Vital sign level I or II criteria	107 (37.2)	145 (50.3)	36 (12.5)
	ISS > 15	20 (20.0)	3 (12.0)	3 (12.0)
	Injury-type level I criteria	41 (41.0)	49 (49.0)	10 (10.0)
	ISS > 15	8 (80.0)	2 (20.0)	0 (0)
	Injury-type level I or II criteria	8 (20.0)	30 (75.0)	2 (5.0)
	ISS > 15	2 (100)	0 (0)	0 (0)
	Mechanism of injury level I or II criteria^a^	66 (24.8)	176 (66.2)	24 (9.0)
	ISS > 15	6 (66.7)	3 (33.3)	0 (0)

Central Netherlands region: the following variables were multiply imputed: systolic blood pressure in 7.0%, respiratory rate in 6.5% and Glasgow Coma Scale in 4.6% of the adult trauma patients

Brabant region: multiple imputation was used for systolic blood pressure in 16.7%, respiratory rate in 28.8% and Glasgow Coma Scale in 4.2% of the adult trauma patients

*LPA* National Protocol of Ambulance Services, *ISS* Injury Severity Score

^a^Mechanism of injury criteria indicate transport to either level I or II trauma centres; no separate criteria in this triage protocol exist that indicates transport to a level I trauma centre

Among the paediatric patients, the compliance rate to the whole triage protocol was 75.3% and 63.1% for the level I criteria (Table 3). Only 26.9% of the severely injured paediatric patients met one or more of the level I criteria; however, 80.0% of the severely injured paediatric patients were transported to a level I trauma centre. In the group of severely injured paediatric patients not meeting any of the level I criteria, 78.9% were transported to a level I trauma centre.

**Table 3:** Distribution of paediatric trauma patients

Region	Criteria	Level I	Level II	Level III
Central Netherlands (*n* = 594)	LPA level I criteria	41 (63.1)	13 (20.0)	8 (12.3)
	ISS > 15	6 (85.7)	0 (0)	1 (14.3)
	LPA level I or II criteria	139 (63.9)	26 (11.9)	41 (18.7)
	ISS > 15	14 (93.3)	0 (0)	1 (6.7)
	Vital sign level I criteria	31 (77.5)	6 (15.0)	3 (7.5)
	ISS > 15	6 (100)	0 (0)	0 (0)
	Vital sign level I or II criteria	76 (65.5)	21 (18.1)	19 (16.4)
	ISS > 15	9 (100)	0 (0)	0 (0)
	Injury-type level I criteria	16 (53.3)	9 (30.0)	5 (6.7)
	ISS > 15	1 (50.0)	0 (0)	1 (50)
	Injury-type level I or II criteria	18 (54.5)	9 (27.3)	6 (18.2)
	ISS > 15	1 (50.0)	0 (0)	1 (50.0)
	Mechanism of injury level I or II criteria^a^	81 (72.3)	13 (11.6)	18 (16.1)
	ISS > 15	9 (100)	0 (0)	0 (0)
Brabant (*n* = 976)	LPA level I criteria	68 (38.0)	94 (52.5)	17 (9.5)
	ISS > 15	1 (100)	0 (0)	(0)
	LPA level I or II criteria	85 (38.1)	117 (52.5)	21 (9.4)
	ISS > 15	1 (100)	0 (0)	0 (0)
	Vital sign level I criteria	66 (38.2)	91 (52.6)	17 (9.8)
	ISS > 15	1 (100)	0 (0)	0 (0)
	Vital sign level I or II criteria	–	–	–
	ISS > 15			
	Injury-type level I criteria	3 (50.0)	3 (50.0)	0 (0)
	ISS > 15	1 (50.0)	1 (50.0)	0 (0)
	Injury-type level I or II criteria	8 (47.1)	8 (47.1)	1 (5.9)
	ISS > 15	1 (50.0)	1 (50.0)	0 (0)
	Mechanism of injury level I or II criteria^a^	16 (39.0)	22 (53.7)	3 (7.3)
	ISS > 15	1 (100)	0 (0)	0 (0)

Central Netherlands region: the following variables were multiply imputed: systolic blood pressure in 41.1%, respiratory rate in 6.2%, paediatric trauma score in 12.8%, and Glasgow Coma Scale in 6.2% of the paediatric trauma patients

Brabant region: multiple imputation was used for systolic blood pressure in 58.3%, respiratory rate in 39.1%, and Glasgow Coma Scale in 7.5% of the paediatric trauma patients. The paediatric trauma score missed in all patients

*LPA* National Protocol of Ambulance Services, *ISS* Injury Severity Score

^a^Mechanism of injury criteria indicate transport to either level I or II trauma centres; no separate criteria in this triage protocol exist that indicates transport to a level I trauma centre

#### Brabant region

A total of 6859 adults and 976 paediatric trauma patients were transported with highest priority by EMS providers in Brabant (Table 1). In total, 165 (2.4%) adult patients and 2 (0.2%) paediatric patients were severely injured.

The compliance rate to the whole protocol was 67.2% for adult trauma patients and 41.8% for the level I criteria (Table 2). The level I triage criteria identified 35.8% of the severely injured adult patients, still, 72.7% were transported to a level I trauma centre. Among the severely injured adult patients not meeting any triage criteria, 63.2% were transported to a level I trauma centre. In this region, the compliance rate to the level I criteria was higher for elderly patients (> 75 years old): 45.9%, compared to 41.1% for young adults.

For paediatric patients, the compliance rate to the whole triage protocol was 48.0% and 38.0% for the level I criteria (Table 3). Both severely injured paediatric patients were transported to a level I trauma centre. One (50.0%) met more than one of the level I criteria and the other did not meet any of the level I criteria.

### Survey analysis

#### Responders and background

In total, 60 EMS providers from Central Netherlands and 48 EMS providers from Brabant filled out the questionnaire. The years of experience ranged from less than a year to 30 years (mean 10 years, standard deviation 7.3). Almost all EMS providers (95.0%) were familiar with the triage protocol. In Central Netherlands, the levels of the trauma centres within the region were well known by most responders. However, in Brabant one-third of the EMS providers did not know the level of 4 of the 11 level II or III trauma centres. Almost all knew which hospitals were level I trauma centres.

#### Factors influencing choice of hospital

How the patient is received by the hospital had more influence on the choice of hospital, than how the EMS providers are received as a professional.

#### Factors influencing choice of level trauma centre

**Fig. 2:**
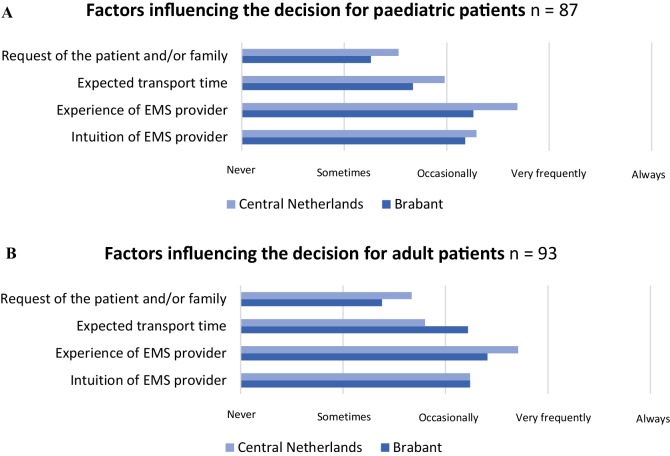
Factors influencing the destination decision for adult and paediatric patients

In the assessment of the patient, the type of injury was the most influential factor when deciding to transport an adult or paediatric patient to either a level I or lower level trauma centre (Fig. 2). Age had the least influence on the destination decision (Table 4). However, the EMS providers did report that they were more easily inclined to transport paediatric patients to a level I trauma centre, compared to adult patients.

**Table 4:** Factors influencing the destination decision from most to least important

	1 (%)	2 (%)	3 (%)	4 (%)	5 (%)
Mechanism of injury					
Paediatric patients	12.6	20.7	55.2	8.0	3.4
Adult patients	7.5	21.5	59.1	9.7	2.2
Vital signs					
Paediatric patients	26.4	32.2	21.8	19.5	0
Adult patients	24.7	43.0	25.8	5.4	1.1
Injury characteristics					
Paediatric patients	55.2	36.8	12.6	1.1	5.7
Adult patients	55.9	29.0	6.5	3.2	5.4
Age					
Paediatric patients	5.7	10.3	10.3	67.8	5.7
Adult patients	1.1	5.4	6.5	76.3	15.9
Other^a^					
Paediatric patients	11.5	0	0	3.4	85.1
Adult patients	15.9	1.1	2.2	5.4	80.6

Paediatric patients: 87 responders, adult patients: 93 responders

^a^Emergency medical service providers reported the wish of the patients of family as other influencing factors for both adult and paediatric patients

Also, EMS provider experience was reported to play an important role in the decision between a level I and lower level trauma centre.

#### Factors influencing undertriage

**Fig. 3:**
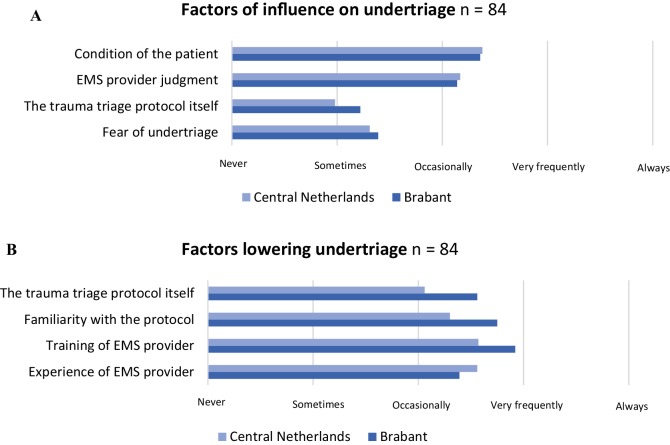
Factors of influence on undertriage and factors lowering undertriage

The training of the EMS providers was reported as most contributory to prevent undertriage (Fig. 3a). In Central Netherlands, it was reported that EMS provider experience could frequently prevent cases of potential undertriage (Fig. 3b); however, EMS provider judgment could also increase undertriage. The triage protocol itself was reported as occasionally capable to prevent undertriage. In Brabant, EMS provider experience was thought to be occasionally capable to prevent undertriage. The long distance to the level I trauma centres was mentioned as cause of undertriage in Brabant.

#### Factors influencing overtriage

**Fig. 4:**
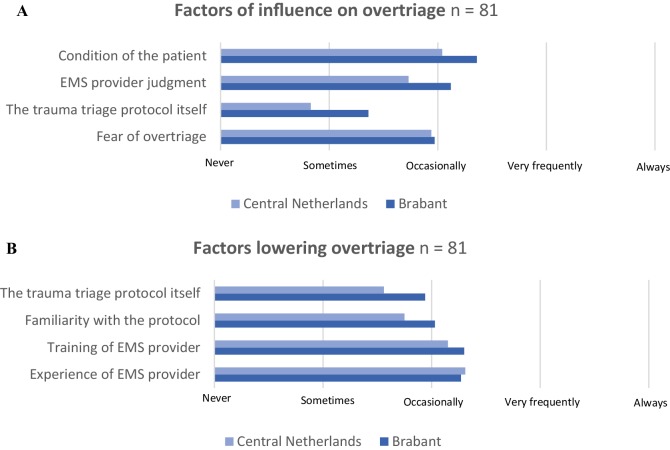
Factors of influence on overtriage and factors lowering overtriage

Experience and training of the EMS provider, familiarity with the triage protocol and the protocol itself were all scored as factors that were reported as occasionally of influence to prevent overtriage (Fig. 4a). The EMS providers suggest that the fear of undertriage in less-experienced EMS providers results in an increased amount of overtriage (Fig. 4b).

#### Consequences of undertriage and overtriage

In both regions, undertriage and overtriage were considered mostly as a learning opportunity. However, 30% of the responders reported that they felt a mistake was made in cases of undertriage. Cases of undertriage are sometimes discussed, whereas cases of overtriage are rarely discussed.

#### Need for adjustment of protocol

According to approximately 90% of the respondents, the current triage protocol does not lack criteria and 95% reported none of the criteria should be removed. Suggestions for adjustment of the triage protocol were: addition of criteria specific for elderly patients and removal of the Revised Trauma Score.

## Discussion

In this study, the compliance to the triage protocol was analysed and EMS providers were surveyed, to gain more insight in the role of EMS provider judgement in the decision-making process of prehospital trauma triage. The compliance rate for adult patients to the level I criteria of the triage protocol was 72% in Central Netherlands and 42% in Brabant. The compliance rate to the level I triage criteria for the paediatric patients was 63% in Central Netherlands and 38% in Brabant. The triage protocol only identified 36% of the severely injured adult patients (ISS > 15). Still, 68% and 63% of the severely injured adult patients were transported to a level I trauma centre in Central Netherlands and Brabant, respectively.

Previously, compliance rates between 40% and 88% have been reported for different triage protocols in different countries and regions [21–24]. In this study, the compliance to the level I triage criteria for adults differed about 30% between the two regions. The EMS providers were surveyed to explore reasons for non-compliance. The questionnaire showed that geographical distance in Brabant can play an important role in the decision-making process. In this region, the nearest hospital is often a level II or III trauma centre; transport of severely injured patients to these trauma centres results in an increase in undertriage. Previous studies reported a lowered likelihood of transport to a higher level trauma centre with increased geographical distance [25–27]. Unfortunately, in the current study, information on distance was not available, so the association between distance and the compliance rate could not objectively be analysed.

EMS providers can choose to deviate from the triage protocol for multiple reasons: EMS provider expertise, experience and familiarity with the triage protocol [17, 28–32]. Compliance and triage quality might improve with feedback to EMS providers on decision-making. In most countries, the EMS providers cannot obtain information from the hospital on specific patients when the EMS medical care is finished due to privacy regulations. Consequently, the EMS providers do not get the feedback they need to learn from possible mistakes. Additionally, involvement of EMS providers in the development of a triage protocol might increase compliance to the triage protocol [17]. When EMS providers believe the triage protocol functions well, they are more inclined to comply with the triage protocol.

Previous studies show that field triage and compliance varies among age groups [22, 33–35]. Triage criteria are less sensitive for paediatric patients; however, the EMS are more easily inclined to transport a paediatric patient to a level I trauma centre, compared to adult patients [24]. The elderly patients, on the other hand, are notoriously undertriaged [13, 19, 34, 36, 37]. Injuries in elderly patients are increasing in frequency, are difficult to recognise—due to a difference in mechanism of injury and masked physiologic derangement—and carry a higher mortality rate compared to the young [22]. Additionally, previous studies report a lower compliance rate for elderly patients [22, 33, 34]. Reported reasons for the transport of elderly trauma patients to lower level trauma centres according to EMS providers were: lack of training, unfamiliarity with the protocol and a feeling that it is not worth to spend expensive trauma centre recourses on elderly patients [29, 30, 34]. Unfortunately, our questionnaire did not focus on elderly trauma patients as a separate group.

According to the EMS providers, the injury-type criteria of the triage protocol had the most influence on the decision between a level I and lower level trauma centre. Among the categories of the triage protocol, the compliance rate was highest for injury-type criteria and lowest for vital sign criteria, in both regions. The injury-type category represents criteria with obvious injuries, easily recognised and clearly indicating transport to a higher level trauma centre [23, 29, 34]. Vital signs, on the other hand, are less apparent: these differ between age groups and might improve during transport, altering the decision for destination facility. The EMS providers reported that the vital sign criteria did influence the destination decision, but to a lesser extent. Previous studies have shown a lower compliance rate to the vital sign criteria [23, 34, 38]. This could be because the majority of the trauma patients have normal or near normal vital signs [39–41].

Most EMS providers reported that additional criteria or removal of criteria would not be necessary. However, the objective analysis of the compliance rates showed that EMS providers often do not adhere to the triage protocol, especially not to the level I criteria. In this study, only a minority of the severely injured patients were identified by the triage protocol. A recent literature review showed that on a worldwide scale, the different triage protocols are not capable to accurately discriminate between patients with and without severe injuries [14]. Thus, efforts to improve the triage protocol are necessary. The current triage protocols used worldwide are outdated and static flow-charts; prediction model with prehospital variables could predict the chance that the patient is severely injured. This prediction model could be integrated in a mobile app, so the EMS provider can calculate the risk of a severe injury quickly and more accurately on-scene. Triage tools integrated in a mobile app are increasingly being developed and used in the prehospital process [42, 43]. The prediction model would include predictors of a severe injury such as age, vital signs, mechanism of injury and injured body regions. As elderly patients are more often undertriaged and all are predictors of severe injury [13, 33, 34, 36, 37, 44–49].

This study has several limitations. First, the response rate to the questionnaire was relatively low; 29%. Previous questionnaire studies showed similar response rates among EMS providers [50–52]. As shown by the range in years of experience among the responders, the results are expected to be representative for all EMS providers of both regions. Additionally, as with all questionnaire studies, an information bias could be introduced; the EMS providers could have given politically correct answers, feeling as if these were expected of them. The response to the questions of the questionnaire was anonymous to minimise this bias as much as possible. Second, for both regions, missing data were present in some variables of the triage protocol. For Central Netherlands, all missing variables could be multiply imputed, limiting the effect on compliance rates. For Brabant, most could be multiply imputed, except for the paediatric trauma score, as it was missing in all paediatric patients. This variable could not be incorporated in the calculation of the compliance rate for paediatric patients. Also, the ISS was only available for the patients who were admitted or who deceased before admission, in the Brabant region. A previous study showed all the severely injured patients (ISS > 15) were admitted or deceased before admission [19]. Accordingly, for Brabant, it was assumed that all the patients discharged from the ED had an ISS < 15. Another limitation is that the compliance rate could be an underestimation because some patients might have been transported to the nearest trauma centre due to life-threatening haemorrhage or acute deterioration. Unfortunately, the data on this were not available. However, a previous study executed in the Netherlands reported only 0.1% of the patients were transported to the nearest trauma centre due to acute deterioration [11]. Additionally, the triage protocol was retrospectively applied based on vital signs and description of the injury and mechanism of injury. The investigators were blinded for destination hospital and the ISS. Last, no data were available to assess the influence of the Helicopter Emergency Medical Services (HEMS) on the choice of hospital.

Quality of prehospital trauma triage is dependent on the accuracy of and compliance to the protocol. The triage protocol functioned poorly; even flipping a coin would provide a better chance of correctly identifying a severely injured patient. Therefore, improvement of the triage protocol should be of first concern. With an accurate protocol, that the EMS providers can trust, the compliance rate may increase. Future studies should additionally focus on quantifying EMS provider judgment to give more insight in reasons for deviating from the triage protocol. Including EMS provider judgment might improve the quality of the triage protocol and compliance rates even more. This might be the solution to get the right patient to the right hospital and improve chances of survival and avert life-long disabilities.

## Conclusion

The compliance rate to the level I criteria varied between 38% and 72% for paediatric and adult patient in the two regions. Despite the fact that only a minority of the severely injured patients were identified by the triage protocol, a large part was transported to a level I trauma centre. Still, the undertriage rate was up to 27%, so improvement is necessary. The triage protocol and triage quality desperately need improvement. A newly developed triage protocol, including EMS provider judgment, serves as an important first step on the read ahead to optimise prehospital trauma triage.
